# Deficiency of Caveolin-1 Aggravates Cardiac Infarction Injury by Disturbing the Endothelial Homeostasis

**DOI:** 10.7150/ijms.101074

**Published:** 2025-01-21

**Authors:** Chunlei Liu, Junwei Zhang, Bing Qi, Zhuqing Li, Qi Li, Li Wang, Chao Li, Ziwei Wang, Zhi Qi, Chengzhi Lu

**Affiliations:** 1 School of Medicine, Nankai University, Tianjin 300071, China.; 2 Department of Molecular Pharmacology, School of Medicine, Nankai University, Tianjin 300071, China.; 3 Department of Cardiology, Tianjin First Central Hospital, Tianjin 300192, China.; 4 Department of Cardiology, The First College of Clinical Medical Sciences, China Three Gorges University, Yichang 443003, China.

**Keywords:** Caveolin-1, Endothelial Homeostasis, Myocardial Infraction

## Abstract

**Objective:** Caveolin-1 (Cav-1) plays a crucial role in maintaining the homeostasis of vascular endothelium. Endothelial dysfunction is involved in many ischemic diseases. However, the role of Cav-1 in myocardial infarction (MI) has not been fully elucidated. Here, this study aims to delineate the function of Cav-1 in MI injury and its impact on endothelial homeostasis.

**Methods:** To elucidate the role of Cav-1 in MI* in vivo,* we generated the global knockout Cav-1 (Cav-1-KO) mice. And we manipulated the expression of Cav-1 by siRNA *in vitro* to evaluate the effects apoptosis, inflammatory response and oxidative stress as well as autophagy flux under the hypoxic model of endothelial cells (ECs).

**Results:** Initially, we found that Cav-1 was mainly expressed in vascular endothelial cells of myocardium. Interestingly, we found that Cav-1 deficiency significantly amplified the size of myocardial infarction areas, alongside the deteriorated cardiac function *in vivo*. Consistently *in vitro*, siRNA-mediated knockdown of Cav-1 exacerbated the endothelial apoptosis, inflammatory response and oxidative stress as well as eliminated autophagy flux. However, pretreatment with β-cyclodextrin (β-CD), which depletes membrane-bound cholesterol and disrupts lipid rafts, markedly mitigated the effects induced by downregulation of Cav-1, respectively.

**Conclusion:** Collectively, in this study, we demonstrated that Cav-1 acts as a protective regulator of MI injury through maintaining endothelial homeostasis. These findings implied that Cav-1 may be a potential therapeutic target for MI injury.

## Introduction

Cardiac ischemia contributes to the myocardial adaption, injury and necrosis process, causing pathologically adverse myocardium remodeling 1. In particular, acute myocardial infarction (AMI) without the consistent coronary blood perfusion usually results in an irreversible heart damage 1, 2. Thereby, in clinical practice, effective and prompt reconstruction of coronary perfusion is essential to save the vulnerable cardiomyocytes 1, 3, 4. Despite recently a large number of studies have provided favorable evidences to improve the cardiac injury in healing and repairing after MI injury 3, 4. However, the translational manipulations to ameliorate the extent of infraction and enhance the reparative process remain many challenges.

Endothelial cells (ECs) are important components to maintain the normal blood supply in myocardium 5, 6. After the ischemia pathophysiological process, ECs prone to be disintegrated conditions, exacerbating inflammation responses, oxidation stress and cell death, etc 6. Accordingly, dysfunctional ECs can release proinflammatory cytokines, for example, interleukin-6 (IL-6) and vascular cell adhesion molecule-1 (VCAM-1), which promote the recruitment and adhesion of leukocytes from the blood to inflamed ECs 6, 7. The infiltrated leukocyte facilitates the inflammatory responses in the damaged myocardium and increases ischemic injury. Besides, ECs dysfunction has been reported to be a key element of MI, which can mediate the MI prognosis 8, 9. Recently, evidences also indicated that ECs-mediated angiogenesis could attenuate the adverse infraction foci and remodeling after the incidence of MI 10. So that, targeting the ECs could be a promising strategy for preventing MI injury.

Caveolin-1 (Cav-1) is a member of caveolin family, which mainly sequester within endothelial caveolae 11. Cav-1 has a dominant implication in cellular metabolism, such as signal transduction and lipid disorders 11, 12. According to previous studies, Cav-1 can aggravate the cardiovascular diseases by competitive inhibition with endothelial nitric oxide synthase (eNOS), subsequently decrease the production of nitric oxide (NO) 11, 13. To the opposite, a recent study indicates that knockout of Cav-1 could alleviate atherogenesis by suppression low-density lipoprotein deposition to favor the ischemia-related diseases 14. The likeliest mechanism appears to be associated with the deregulation of endothelial inflammation and independent of the suppressed eNOS. It is documented that MI injury patterns can activate inflammatory pathways at the early phase and promote the ischemic insults 15. Therefore, regulation of the ECs function via Cav-1 may be a significant target to MI injury. However, the underlying mechanism by how Cav-1 maintains endothelial homeostasis has not been thoroughly demonstrated.

In this study, we investigated the role of Cav-1 in MI injury and endothelial homeostasis under hypoxia condition. By using Cav-1 knockout mice (Cav-1-KO), we demonstrated that knockout of Cav-1 aggravated MI injury and cardiac dysfunction after MI. Furthermore, by cultivating ECs and utilizing glucose-oxygen deprivation (OGD) injury *in vitro* models, we observed that Cav-1 was markedly responsible for cell survival, inflammatory response, oxidative status as well as autophagy flux when ECs were under hypoxia injury. And pharmacological destruction of the caveolae by β-cyclodextrin (β-CD) further elucidated that the function of Cav-1 in ECs was dependent on the caveolae. In conclusion, these results reveal that Cav-1 may be serve as an important therapeutic target for MI.

## Materials and Methods

### Reagents and antibodies

JC-1 (cat#C2003S) for probing the mitochondrial membrane potential assay kit were purchased from Beyotime Biotechnology Institute (Shanghai, China). The L-NAME (HY-18729A) was from MedChemExpres (MCE, USA). And 2,3,5-Triphenyl tetrazolium chloride (TTC, cat#G3005) used for detecting the infarct foci was obtained from Solarbio (Beijing, China). Annexin V-fluorescein isothiocyanate (FITC)/propidium iodide (PI) apoptosis assay kit (cat#40302ES50) used to measure apoptosis was purchased from YEASEN (Shanghai, China). Reactive Oxygen Species Assay Kit (cat#S0033S, Beyotime) was used to detect the reactive oxygen species levels. The β-cyclodextrin (cat#C8511) for deconstruction of caveolae was obtained from Solarbio (Beijing, China). The primary antibodies anti-Caveolin-1 (cat#3267S), anti LC3 (cat#2775s), anti P62 (cat#39749s), anti SOD2 (cat#13141S), anti P65 (cat#8242S) and anti-phospho-P65 (Ser) (cat#3033S) used for western blotting and/or immunofluorescence were purchased from CST (Cell Signaling Technology, Danvers, Essex County, MA, USA); anti GAPDH (cat# A19056) were purchased from ABclonal (Wuhan, China); anti Nrf2 (cat#SAB5700720) were purchased from Sigma (Aldrich, USA); anti CD31 (cat#40302ES50) and anti Cav-1(cat#40302ES50) used for immunofluorescence were purchased from Santa Cruz Biotechnology (Santa Cruz, Calif); anti Anti-Cardiac Troponin T antibody (cTnT, cat#ab8295) was obtained from Abcam (Cambridge, Mass). Secondary antibodies for rat (cat#ZF-0315) and rabbit (cat#ZF-0316) species were obtained from ZSGB-BIO (Beijing, China).

### Animals and animal models

Genetically global knockout Cav-1 mice were obtained from Shanghai Model Organisms Center Inc. (Shanghai, China). C57BL/6J mice (6-8 weeks of age) were purchased from SPF biotechnology (Beijing, China). All the animals were housed in Nankai Animal Resources Center with ethic committee approval. The optimate animals underwent MI injury as previous description 16. Briefly, the mice were anesthetized using 1.5% isoflurane and kept in a supine position. When the left axilla was surgical exposure, the left fourth intercostal was cut. Then the heart was exposed, following the left anterior descending coronary artery (LAD) permanently ligated by a slipknot (6-0 silk). For the sham group, mice suffered the same procedures but no tying the slipknot. Ischemic myocardial tissue was turning to be pale, which was confirmed as effective models. After 48h of ligation, ultrasound M-mode echocardiography was performed to detect the cardiac function. Subsequently, the heart and corresponding samples were harvested.

### Small interfering RNA (siRNA) transfection

The siRNA sequence targeting Cav-1 was synthesized by GenePharma (Shanghai, China). Briefly, when cells reached 70% density, Cav-1 and scramble siRNA (20nM) separately mixed with Transfection Reagent (GenePharma, China) and added into the cells. The medium was replaced with normal DMEM after 6h transfection. The efficiency of knockdown was assessed by qRT-PCR and Western blotting. The siRNA sequences are as following:

Cav-1 siRNA: Forward (5′-3′): CCUUCACUGUGACGAAAUATT; Reverse (5′-3′): UAUUUCGUCACAGUGAAGGTT; Scramble siRNA: Forward (5′-3′): UUCUCCGAACGUGUCACGUTT; Reverse (5′-3′): ACGUGACACGUUCGGAGAATT.

### OGD injury model *in vitro*

To mimic ischemic model *in vitro*, human umbilical vein endothelial cells (HUVECs) were cultured in glucose-free Dulbecco's Modified Eagle Medium (DMEM) (Procell, China, cat#PM150270) and sustained in AnaeroPack anaerobic system with 5% CO_2_ and 95%N_2_ at 37°C for 90min.

### Western blot analysis

Cells were lysed via PMSF (1mM) mixed with RIPA buffer. Proteins were extracted by centrifugation (12,000 rpm) for 30 min. Supernatant were boiled with loading buffer for 10min. And then, the mixture was separated by SDS/PAGE and transferred to PVDF membranes. The membranes were blocked with 5% non-fat milk-TBST buffer and incubated with primary antibodies at 4°C overnight. Later these membranes were rinsed with TBST buffer and incubated with horseradish peroxidase (HRP) conjugated secondary antibody (1:5000) at RT for 1h. The blots were detected by the enhanced chemiluminescence (ECL) solution. And the bands were analyzed by Image J software (Bethesda Md, USA).

### Reverse transcription quantitative real time polymerase chain reaction (qRT-PCR)

The RT-PCR experiment was described previously. Total RNA was isolated using Trizol reagent (Solarbio, China). To assessment the RNA quality and concentration, the absorbance ratio 260/280 nm was calculated by a NanoDrop device (Thermo Fisher Scientific). The equal amount of mRNA was converted into cDNA by a Reverse Transcription Kit (Yeasen, China, cat#11141ES60). Firstly, we utilized 5×g DNA Digester Mix to remove the genomic DNA. Thereafter, 4× Hifair III SuperMix was incorporated to assemble the reverse transcription system. Then, the mix was reacted under a standardized procedure according to the manufacturers' instructions. The qRT-PCR was performed using SYBR Green Master Mix (Yeasen, China) with CFX96 RT-PCR System (Bio-Rad, USA). The results were normalized against β-Actin. Sequences of primer are following:

Cav-1 forward: 5′-CCGTAGTGGGTATGGTTGA-3′; Cav-1 reverse: 5′-GTTGATGAGAGGCAGGGA-3′; SOD2 forward: 5′-CACCGAGGAGAAGTACCAGGAG-3′; SOD2 reverse: 5′-CCACCACCGTTAGGGCTGAG-3′; Nrf2 forward: 5′-AGTCCAGAAGCCAAACTGACAGAAG-3′; Nrf2 reverse: 5′-GGAGAGGATGCTGCTGAAGGAATC-3′; ICAM-1 forward: 5′-ACCTATGGCAACGACTCCTTCTC-3′; ICAM-1 reverse: 5′-GTGTCTCCTGGCTCTGGTTCC-3′; VCAM-1 forward: 5′-ATCCTCCTTAATAATACCTGCCATTGG-3′; VCAM-1 reverse: 5′-TCTGTGCTTCTACAAGACTATATGACC-3′; MCP-1 forward: 5′-CCAGCAGCAAGTGTCCCAAAG-3′; MCP-1 reverse: 5′-TGCTTGTCCAGGTGGTCCATG-3′; Ccl5-1 forward: 5′-CCCTGCTGCTTTGCCTACATTG-3′; Ccl5-1 reverse: 5′-AAGACGACTGCTGGGTTGGAG-3′; β-Actin forward: 5′-GGCCAACCGCGAGAAGATGAC-3′; β-Actin reverse: 5′-GGATAGCACAGCCTGGATAGCAAC -3′.

### Measurement of reactive oxygen species (ROS)

In order to measure the ROS production, the cellular dishes were washed for three times and incubated in DHE solution (10uM) for 20min at 37°C. After then the dishes were washed again and pictured via fluorescence microscope (Olympus, Japan). Analysis was performed by Image J software (Bethesda Md, USA). Besides, ROS Assay Kit was used to detect ROS levels according to the specific instructions and analyzed by flow cytometry.

### Measurement of cell apoptosis assay by flow cytometry

Annexin V/PI assay kit was used to detect cell apoptosis. The harvested cellular plates were stained with the corresponding Annexin V-FITC and PI according to the instructions and analyzed by flow cytometry.

### Measurement of Serum CK-MB Isoenzyme and LDH levels

After MI for 48h, blood samples were collected and centrifuged at 3000g for 10min to layer the serum. The serum lactate dehydrogenase (LDH) and creatine kinase MB (CK-MB) isoenzyme levels were indictors of myocardial injury. Plasma concentrations of LDH and CK-MB were performed and analyzed in the BECKMAN COULTER CHEMISTRY ANALYZER AU5800 according to the corresponding reagent protocols.

### Echocardiography

After LAD ligation for 48min, two-dimensional and M-mode echocardiography were performed via Vevo2100 system (Visual Sonics, USA). Parameters of left ventricle interior diameter in diastole (LVIDd), left ventricle interior diameter in systole (LVIDs), left ventricular end-diastolic volume (LVEDV) and left ventricular end-systolic volume (LVESV) were recorded. According to the recorded parameters, left ventricular ejection fraction (LV EF) = (LVEDV-LVESV)/LVEDV and left ventricular fractional shortening (LV FS) = (LVIDd-LVIDs)/LVIDd were calculated.

### TTC staining

The harvested heart was rapidly transferred into PBS for 30 seconds, and then was frozen for 15min at -80°C. Subsequently, the heart was cut into six slices and incubated in 2%TTC solution at 37°C for 15min. After fixed in 4% paraformaldehyde for 24h, it was pictured in time. Under the stereo microscope, pale areas represented infract myocardium, conversely the red areas indicated viable myocardial tissue. Image J software (Bethesda Md, USA) was used to quantitate analysis of the infarct foci.

### Immunofluorescence staining

Heart slices were fixed in 4% paraformaldehyde overnight and permeabilized with Triton X-100. Following the sections (6μm) were blocked with goat serum (5%) for 30min at normal room temperature. Then, the samples were incubated with specific antibodies at 4°C overnight. Subsequently, it was washed extensively and incubated with corresponding secondary antibodies for 1h. It was visualized using microscope (Nikon, Japan). Confocal cell dishes were performed with the same procedures and were captured under a confocal microscope (Olympus FV3000, Japan). Finally, these pictures were quantitatively analyzed through Image J software (Bethesda Md, USA).

### Statistical analysis

All the data are expressed as means±standard deviation (SD) for at least 3 independent experiments. GraphPad Prism 9 Statistics program was unitized for statistical analysis. Student's t-test was used for analyses between two groups, as well as 2-way ANOVA analysis followed by the Dunnett's multiple comparisons test was performed among 3 or more groups. Statistically the value of P<0.05 was defined significant.

## Results

### Cav-1 is expressed in vascular ECs of myocardium

**Figure 1:**
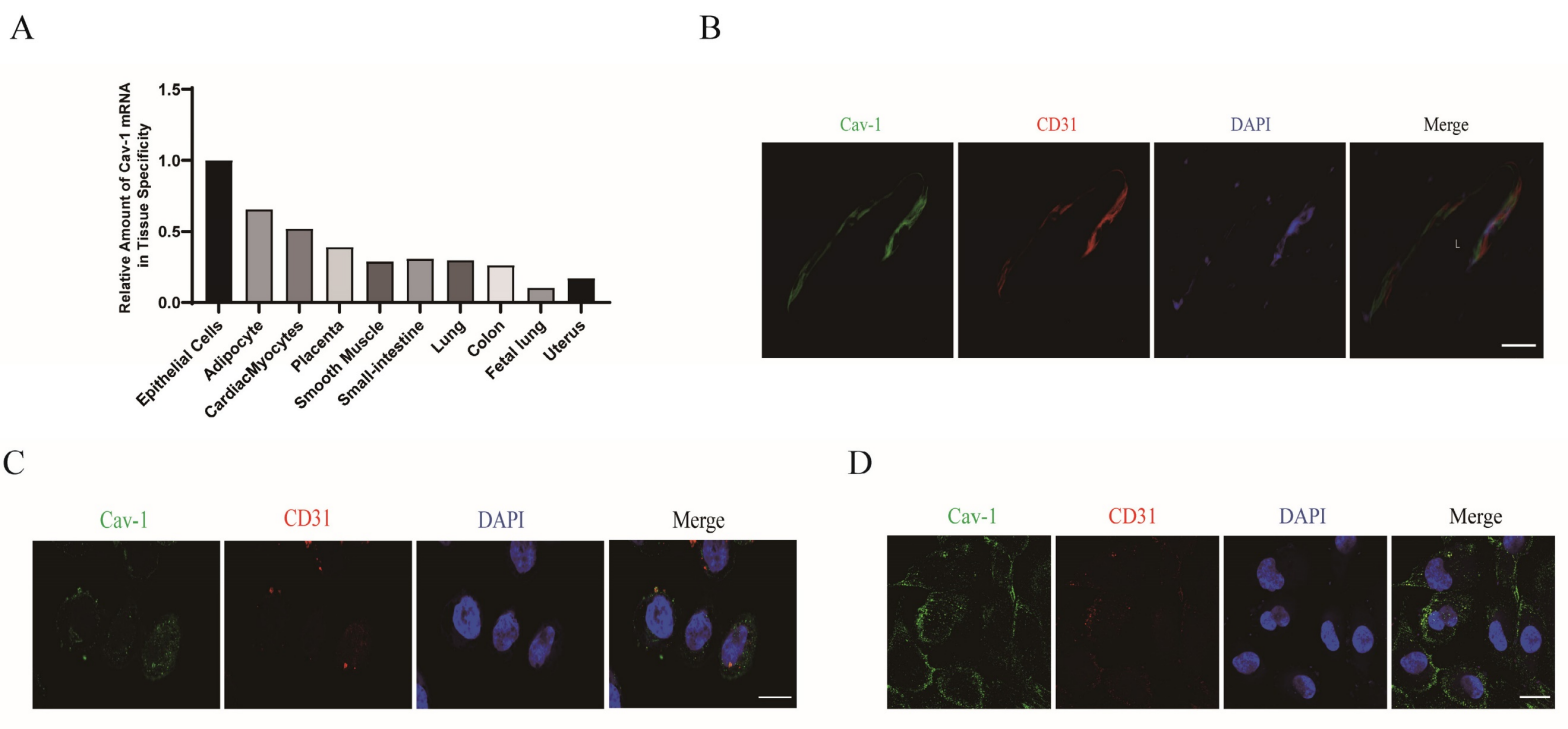
**Cav-1 predominately expresses in vascular ECs of myocardium.** A, Relative analysis of Cav-1 expression in different tissues by BioGPS databases. B, Cardiac sections immunofluorescence staining of Cav-1 (green) and CD31 (red) were performed in adult mouse vascular endothelium. Scale bar, 50μm. C-D, Immunofluorescence staining of Cav-1 (green) and CD31 (red) in HCAEC (human coronary artery endothelial cells, C) and HUVECs (human umbilical vein endothelial cells, D) were performed* in vitro*. DAPI stains nuclei (blue). Scale bar, 20μm. Images were selected based on the average from independent experiments, and the same criteria apply throughout this article.

ECs are crucial for angiogenesis and the revascularization process following cardiac ischemia 17-19. To clarify the role of Cav-1 in MI, firstly we focused on determining the specific localization of Cav-1 protein in the adult heart. An analysis of the BioGPS databases revealed a high expression in epithelial cells (Fig. 1A). And then, we examined the expression of Cav-1 in the hearts of adult mice. Immuno-labeled cardiac cross-sections were obtained to explore the histological distribution of Cav-1 in mice heart. Consistently, immunofluorescence staining showed that Cav-1 was predominantly expressed in vessels (Fig. 1B). Moreover, confocal imaging of Cav-1 was further demonstrated the distribution of Cav-1 protein in ECs (Fig. 1C and D). Taken together, these results suggested that Cav-1 was predominately expressed in ECs.

### Cav-1 deficiency aggravates myocardial ischemia injury in mice

**Figure 2:**
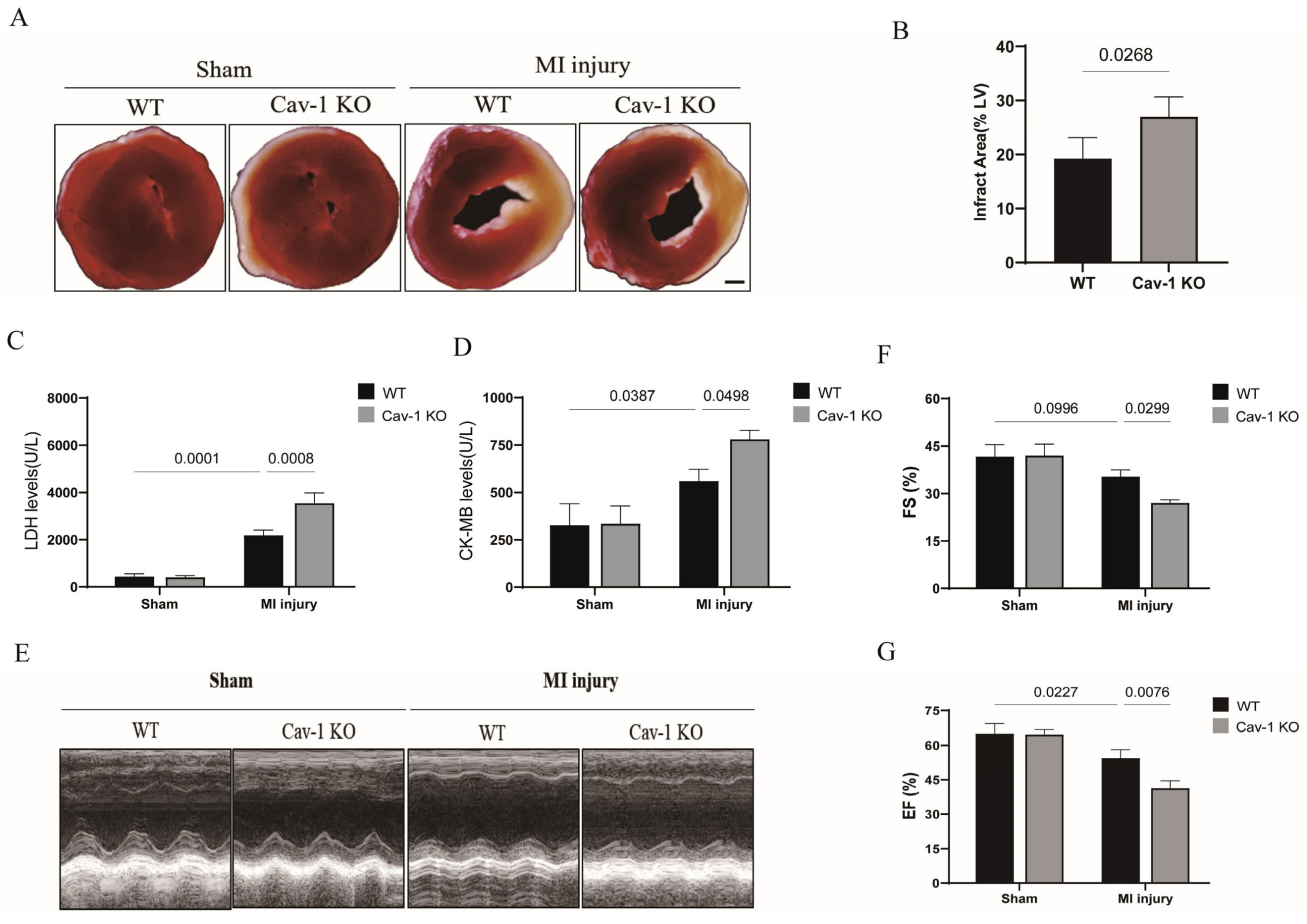
**Cav-1 deficiency aggravates myocardial ischemia injury in mice.** A, Representative images of triphenyl tetrazolium chloride (TTC) staining were shown for each group. Scale bar, 2mm. B, Infarct areas were quantified after myocardial ischemia injury for 48 hours in Cav-1 KO and WT mice (n=3). C-D, The serum lactate dehydrogenase (LDH) levels and creatine kinase MB (CK-MB) isoenzyme levels were analyzed by chemistry analyzer (n=3). E, Representative echocardiograph images were shown for each group. F-G, Fractional shortening (FS%) and ejection fraction (EF%) were analyzed based on the ultrasonographical data (n=4). Data was analyzed using 2-way ANOVA with the Dunnett's multiple comparisons test (B, C, D, F and G). Data were shown as the Mean±SEM, respectively.

To examine the role of Cav-1 in ischemic heart, we generated Cav-1 global knockout mice. Wild-type (WT, Cav-1^+/+^) and Cav-1 knockout (Cav-1-KO, Cav-1^-/-^) mice were raised for 6-8 weeks before being subjected to MI model, respectively. In comparison with WT sham group, WT mice subjected to MI significantly exhibited increased infract size. Furthermore, knockout of Cav-1 after MI injury aggravated infract size when compared with WT-MI mice (Fig. 2A and B). In line with these results, among WT mice, serum LDH levels (Fig. 2C) and CK-MB isoenzyme levels (Fig. 2D) were higher in myocardial infracted group. But myocardial infracted Cav-1-/- mice were detected further elevated serum LDH levels (Fig. 2C) and CK-MB isoenzyme levels (Fig. 2D) when compared with myocardial infracted WT groups. Similar to what was revealed in cardiac damage, we found that myocardial infracted Cav-1-/- mice exhibited the deteriorated cardiac function after the MI injury, prominently as indicated by decrease of EF and FS compared with myocardial infracted Cav-1 +/+, respectively (Fig. 2E - G). Thus, these findings identified that Cav-1 act as a protective factor in MI injured heart.

### Cav-1 knockdown promotes endothelial oxidative stress injury *in vitro*

**Figure 3:**
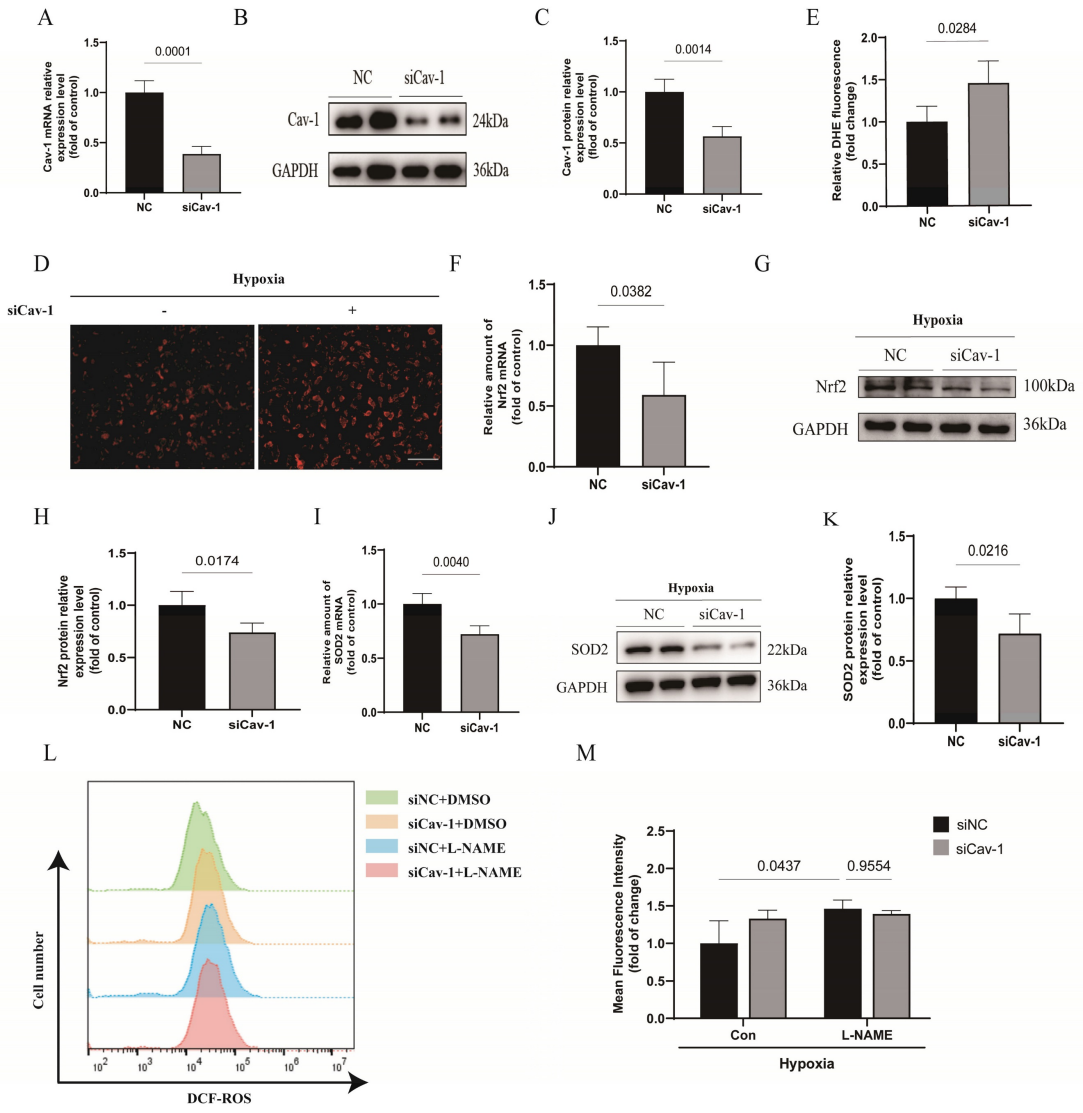
**Cav-1 knockdown promotes endothelial oxidative stress injury *in vitro*.** A-C, Human umbilical vein endothelial cells (HUVECs) were transfected with siRNA (small interfering RNA)-NC (negative control) or siRNA-Cav-1 for 48 h. Immunoblot analysis and reverse transcription quantitative real time polymerase chain reaction (qRT-PCR) were assayed to quantitate the relative knockdown ratio (n=3). D, The representative images of DHE (dihydroethidium) staining were shown. Scale bar, 100μm. E, Positive percentage of DHE staining was quantified (n=4). F-H, The mRNA and protein levels of Nrf2 (nuclear factor erythroid 2-related factor 2) were measured in HUVECs that had been transfected with control siRNA or Cav-1 siRNA (n=3). I-K, The mRNA and protein levels of SOD2 (superoxide dismutase 2) were measured (n=3). L-M, Determination and analysis of ROS upon L-NAME treatments detected by Reactive Oxygen Species Assay Kit based on flow cytometry. Statistical analysis was performed by unpaired Student's* t*-test (A, C, E, F, H, I and K) and 2-way ANOVA with the Dunnett's multiple comparisons test (M). Data were presented as the Mean±SEM, respectively.

To understand the role of Cav-1 in oxidative stress, Cav-1 siRNA was used in following experiments *in vitro*. The mRNA and protein levels of Cav-1 were significantly reduced in HUVECs transfected with Cav-1-siRNA compared to those transfected with negative control (NC) sequences (Fig. 3A-C). We found that knockdown of Cav-1 markedly enhanced ROS accumulation in ECs under hypoxia (Fig. 3D and E). Additionally, both nuclear factor erythroid 2-related factor 2 (NRF2) and superoxide dismutase 2 (SOD2) are important antioxidant proteins 20-22. We observed that Cav-1 siRNA significantly down-regulated the expressions of NRF2 and SOD2 either in mRNA or in protein levels, indicating that knockdown of Cav-1 results in decreased ability of antioxidant system (Fig. 3F-K). Furthermore, in elucidating the regulatory mechanism of oxidative stress, we adopted the L-NAME to inhibit the activity of endothelial nitric oxide synthase (eNOS). Although downregulation of Cav-1 was involved in endothelial oxidative stress, there was no significant difference in oxidative stress when eNOS was inhibited compared with control group which implies that the eNOS was attributable to the endothelial oxidative stress in Cav-1-mediated effects (Fig. 3L-M). Together, these results suggest that Cav-1 plays a role in promoting endothelial oxidative stress injury.

### Knockdown of Cav-1 aggravates hypoxia-induced endothelial apoptosis

**Figure 4:**
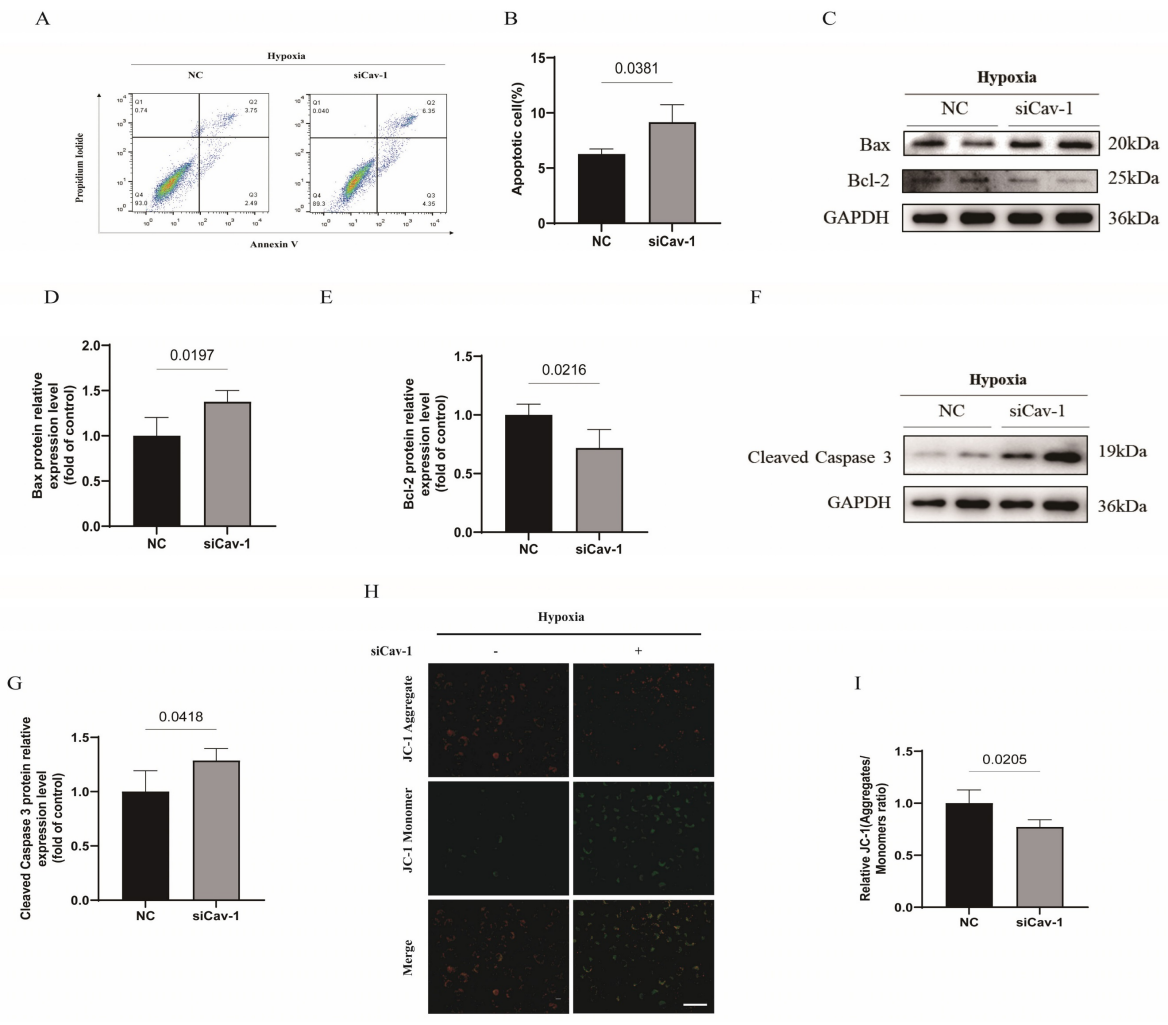
** Knockdown of Cav-1 aggravates hypoxia-induced endothelial apoptosis.** A-B, Annexin V & PI flow cytometry were used to quantitate the proportion of apoptotic cells (n=3). C, The expression of apoptosis-related proteins (Bax and Bcl-2) were determined by Western blot. D-E, The Bax and Bcl-2 proteins expression were quantified (n=3). F, The representative images of immunoblotting of cleaved caspase 3 were shown. G, The expression of cleaved caspase 3 was quantified (n=3). H-I, JC-1 assays were used to detect mitochondrial membrane potential in ECs with or without knockdown of Cav-1 protein (n=3). Scale bar, 100μm. Statistical analysis was assessed by unpaired *t* test (B, D, E, G and I). Data was represented as Mean±SEM.

To confirm the effect of Cav-1 on apoptosis of ECs, we conducted Annexin V/PI flow cytometry test in HUVECs. The results showed an increased apoptosis rate in ECs following the knockdown of Cav-1 (Fig. 4A and B). We found that the apoptosis related protein-Bax was markedly increased in Cav-1 siRNA group (Fig. 4C and D). In contrast, the expression of Bcl-2, an anti-apoptotic protein, was reduced in Cav-1 siRNA group when compared with the NC group (Fig. 4C and E). Besides, hypoxia-elicited caspase-3 activation was augmented under the Cav-1 knockdown status (Fig. 4F and G). In addition, Cav-1 siRNA revealed a notably reduction of mitochondrial membrane potential as confirmed by the JC-1 assay, consistently (Fig. 4H and I). These findings collectively suggest that the knockdown of Cav-1 can amplify hypoxia-induced apoptosis in ECs.

### Knockdown of Cav-1 exacerbates endothelial cell inflammatory responses

**Figure 5:**
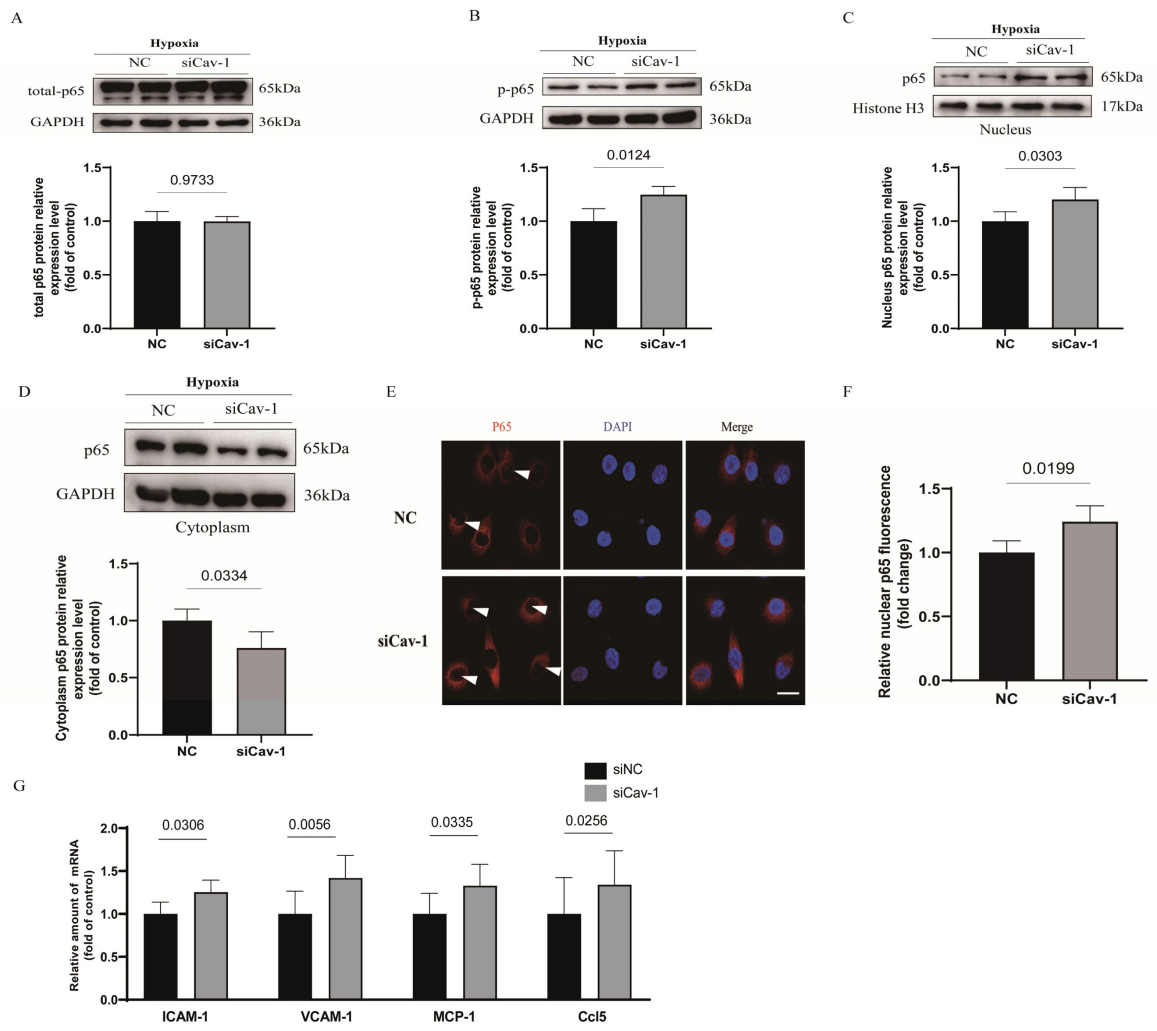
** Knockdown of Cav-1 exacerbates endothelial cell inflammatory responses.** A-B, Expression and quantification of nuclear factor-κB (NF-κB) pathway-related proteins including total p65 and p-p65 in cellular lysates harvested from negative control (NC) and Cav-1-siRNA (siCav-1) groups (n=3). C-D, Cytoplasmic and nuclear fractions from HUVECs with or without Cav-1-siRNA interference were immunoblotted with antibodies against the specific proteins as well as the quantification was shown at the bottom normalized to GAPDH or Histone 3 (n=3). E-F, With the hypoxia injury for 90min, representative images of cellular immunofluorescence staining for p65 (red) and DAPI (blue) with the quantification data (n=4). Scale bar, 20μm. G, The mRNA levels of intercellular cell adhesion molecule-1 (ICAM-1), vascular cell adhesion molecule-1 (VCAM-1), monocyte chemoattractant protein-1 (MCP-1) and C-C motif chemokine ligand 5 (Ccl5) were measured in HUVECs that had been transfected with control siRNA or Cav-1 siRNA before hypoxia injury (n=4). Statistical analysis was performed by unpaired *t* test (A, B, C, D, F and G). Data were shown as the Mean±SEM, respectively.

Cellular nuclear factor-κB (NF-κB) complexes play an important part to modulate the inflammatory homeostasis involved in the release of numerous proinflammatory mediators 23. To assessed the implications of Cav-1 in endothelial inflammatory homeostasis under hypoxia injury, we assayed p65 subunit and subcellular localization of NF-κB. Interestingly, we found that the whole cell lysates of p65 was comparable between the 2 groups with or without transfection of Cav-1-siRNA (Fig. 5A). However, phosphorylation of p65 protein was significantly augmented, which indicates the activation of NF-κB after Cav-1-siRNA transfection (Fig. 5B). Besides, p65 could be trafficked into nuclear to induce the target gene expression. Therefore, we isolated the nuclear and cytoplasmic lysates and found that the proportion of p65 in nuclear was accumulated (Fig. 5C) and in cytoplasm was limited (Fig. 5D) when Cav-1 protein was disrupted. Indeed, immunofluorescence staining further clarify the Cav-1 knockdown increased the co-localization of nuclear compared with NC groups (Fig. 5E and F). Afterwards, we have further detected endothelial inflammatory markers under hypoxia injury, including intercellular cell adhesion molecule-1 (ICAM-1), vascular cell adhesion molecule-1 (VCAM-1), monocyte chemoattractant protein-1 (MCP-1) and C-C motif chemokine ligand 5 (Ccl5), and found upregulated results when Cav-1 protein was knockdown. (Fig. 5G) Thus, we conclude that downregulated of Cav-1 can amplified the endothelial inflammatory responses.

### Downregulation of Cav-1 significantly suppresses the endothelial autophagic flux under the hypoxia injury

**Figure 6:**
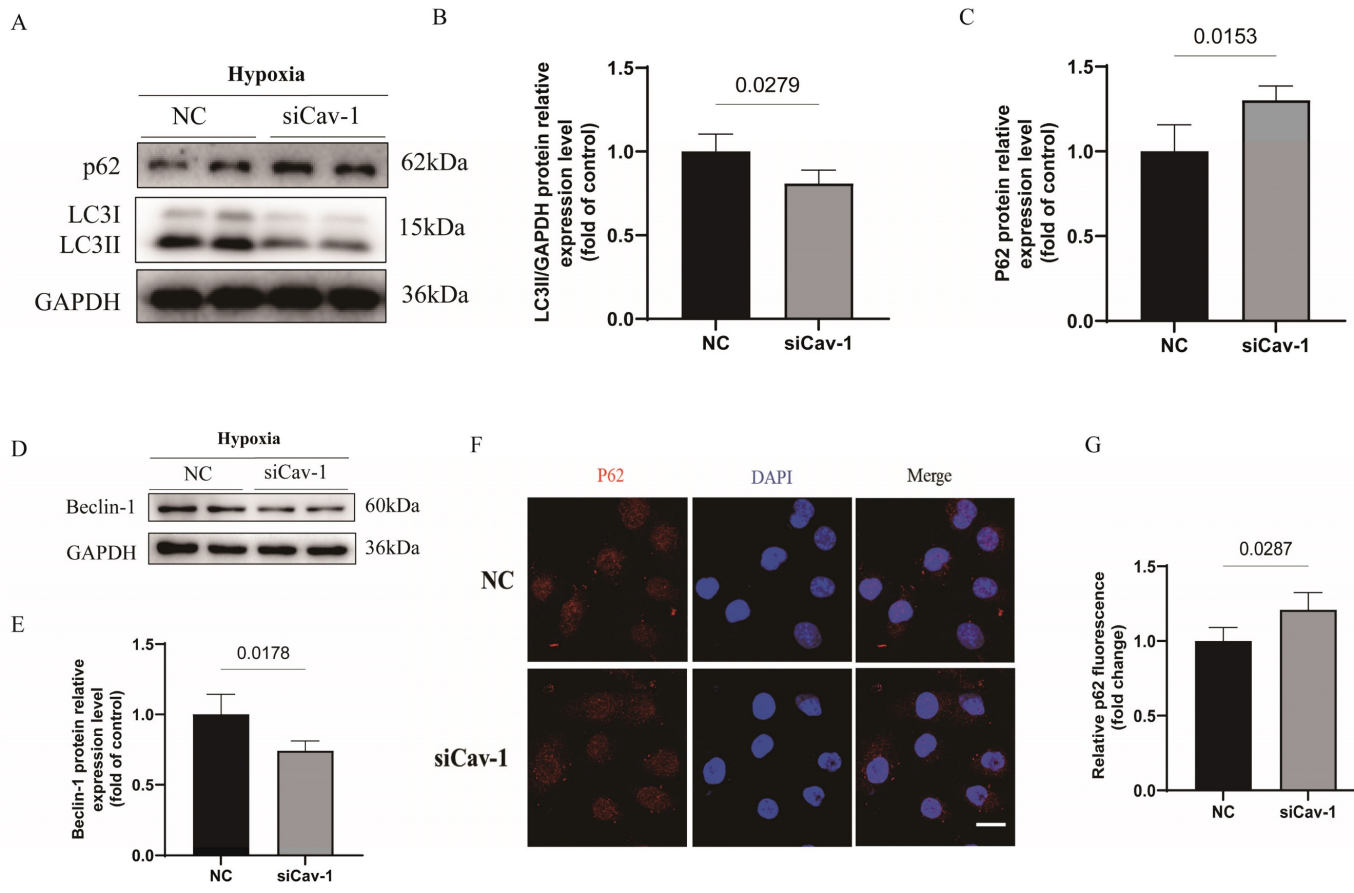
** Downregulation of Cav-1 significantly suppresses the endothelial autophagic flux under the hypoxia injury *in vitro*.** A-C, Immunoblot and quantitative analysis of LC3II and p62 protein levels in negative control (NC) and siRNA-Cav-1 groups of HUVECs lyses (n=3). D-E, Immunoblot and quantitative analysis of Beclin-1 protein levels in HUVECs after hypoxia injury (n=3). F-G, After the hypoxia injury, representative images and quantitative analysis of p62 puncta, respectively (n=3). Scale bar, 20μm. Significant differences were evaluated using unpaired* t* test (B, C, E and G). Data were shown as the Mean±SEM.

It is well-known that activate autophagic flux process can blocked the progressive ischemic injury 24, 25. To investigated the role of Cav-1 in autophagy status, we monitored the autophagic flux in cultured ECs under hypoxia stress. Notably, autophagy was inhibited following inferences of Cav-1 protein, as manifested by a dramatically decrease in LC3II protein levels (Fig. 6A and B) and increase in p62 protein levels (Fig. 6A and C) of WB experiments. In line with the autophagic status, Beclin-1 levels were significantly decreased following Cav-1 knockdown (Fig. 6D and E). In addition, in agreement with the WB assays results, p62 puncta was considerate increase in cells with siRNA-mediated Cav-1 knockdown (Fig. 6F and G). So, these data suggested that Cav-1 deficiency suppressed the endothelial autophagy flux.

### Cav-1-Mediated the endothelial homeostasis dependents on caveola structure in ECs

**Figure 7:**
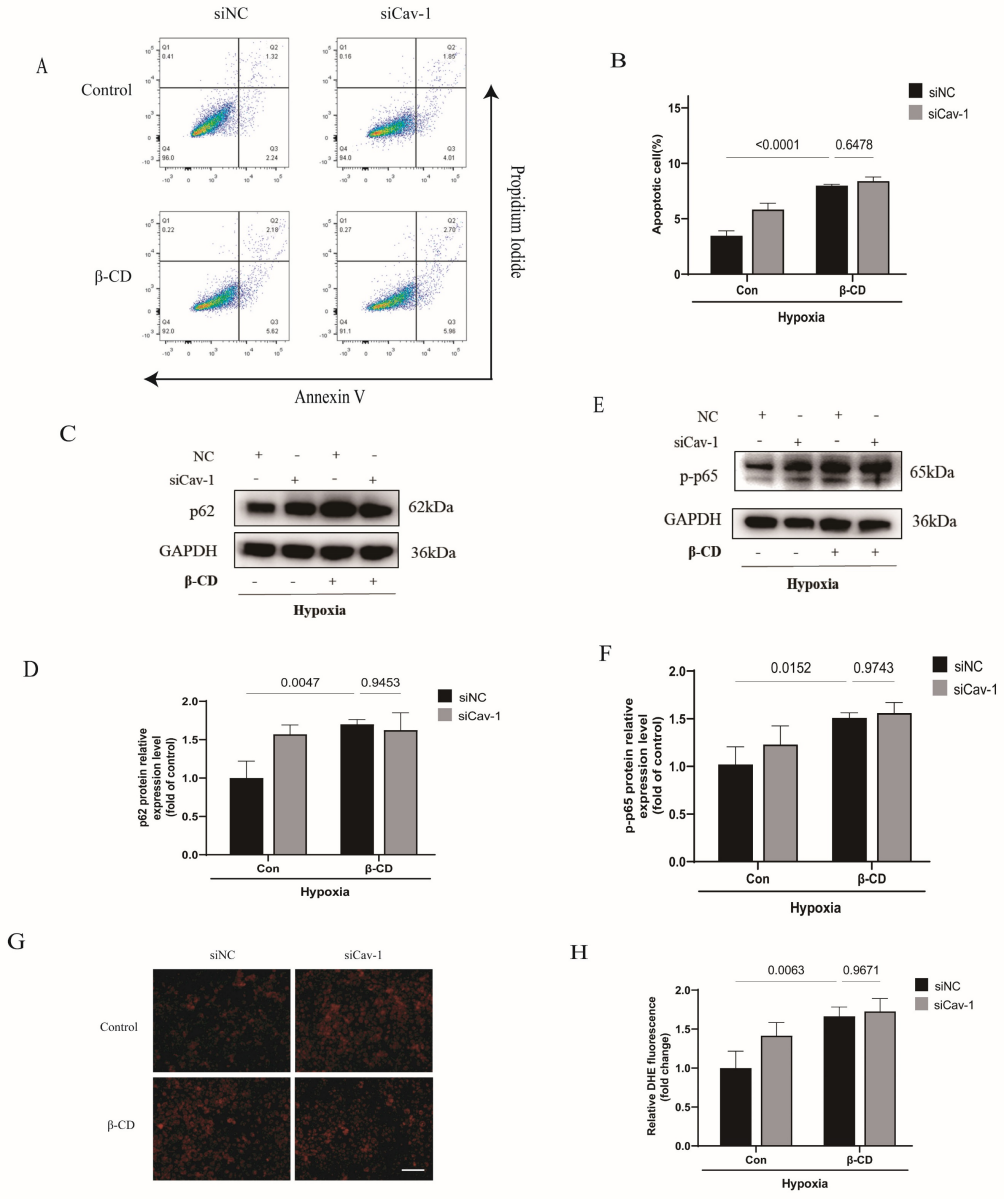
** Cav-1-mediated the endothelial homeostasis dependents on caveola structure in ECs.** HUVECs were transected with Cav-1-siRNA or control sequences for 48h and then pretreated with β-CD (5 mmol/L) for 2h before the hypoxia insults. A, Flow cytometric analysis of HUVECs populations derived from negative control (NC) and Cav-1-knocdown (Cav-1 KD) groups with or without β-CD treatment. B, Flow cytometry-based quantification of apoptotic HUVECs populations (n=3). C-D, Proteins expression and quantification levels of p62 in HUVECs lyses from NC and Cav-1-KD dishes with or without β-CD pretreatment (n=3). E-F, Proteins expressive and quantized levels of p-p65 in HUVECs lyses with or without β-CD pre-interventions (n=3). G, Representative images of DHE staining after the hypoxia stimulation. Scale bar, 100μm. H, The quantification data of DHE staining were shown (n=4). Data was analyzed using 2-way ANOVA with the Dunnett's multiple comparisons test (B, D, F and H). Data were shown as the Mean±SEM, respectively.

Cav-1 is dominatingly located in the intracellular caveola 26. Thus, we hypothesized that Cav-1 may exert its functions through endothelial caveola structure. To test the hypothesis, we pretreated ECs with β-CD to destroy the caveola structure. Interestingly, the effects of Cav-1-siRNA-induced cellular apoptosis were mitigated when ECs were cultured in the presence of β-CD (Fig. 7A-B). Similarly, the knockdown of Cav-1 showed comparable levels of autophagic flux, as indicated by similar p62 protein levels (Fig. 7C-D). Additionally, p-p65 also exhibited comparable levels after interference with Cav-1 proteins under β-CD pretreatment (Fig. 7E-F). Furthermore, DHE staining indicated that the effects of Cav-1 downregulation on oxidative stress were disappeared when it was pretreated with β-CD (Fig. 7G-H). Therefore, these results elucidate that caveola structure is crucial for Cav-1 protein.

## Discussion

**Figure 8:**
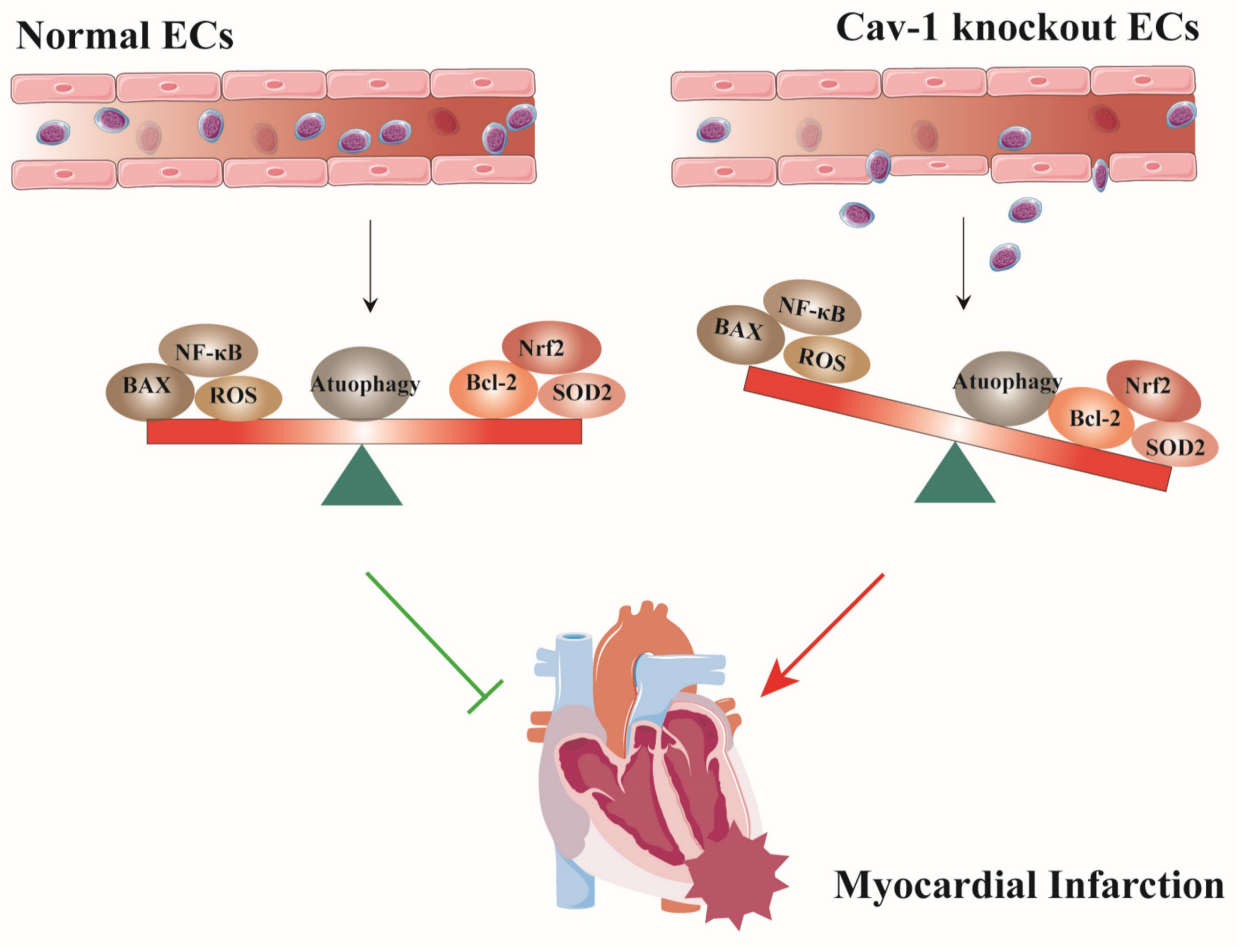
** Schematic representation of the effects of Cav-1 on myocardial infarction (MI).** Cav-1 deficiency exacerbates MI injury by disrupting endothelial homeostasis. This includes promoting endothelial apoptosis, increasing the inflammatory response, enhancing oxidative stress, and impairing autophagic flux.

In this study, we used Cav-1-KO mice and siRNA-mediated knockdown of Cav-1 protein in ECs to demonstrate that the role of Cav-1 in endothelial homeostasis during MI. Deficiency of Cav-1 *in vivo* results in deteriorating cardiac function and progressing infracted foci. Similarly, *in vitro* interferences of Cav-1 protein with siRNA alter the endothelial homeostasis of apoptosis, oxidative stress, inflammatory responses and autophagy flux (Fig. 8). Thus, these findings imply that Cav-1 may be a potential therapeutic target for MI injury.

The pathophysiological process of MI involves of the dysfunction of vascular endothelium in the ischemic myocardium 27. Herein, our experiments preliminarily provide evidences that adult heart highly expressed Cav-1 in endothelium (Fig. 1), suggesting that Cav-1 may be related to the MI injury by regulation of vascular function. Cav-1 is a fundamental member of the mammalian caveolins family 26, 28. Factually, in variety of vascular diseases, Cav-1 was broadly considered as key factor that can influence the pathological outcomes. Endothelial-specific over-expression of Cav-1 attenuated cerebral ischemia by oligodendrogenesis 29, whereas downregulation of Cav-1 promoted cerebral ischemia injury. Besides, down-modulation of Cav-1 aggravated the development of pulmonary hypertension (PH) and congestive heart failure (CHF) after cardiac ischemia injury 30. In line with these results, we found that knockout of Cav-1 promoted myocardial infract zone and deteriorated cardiac function after MI injury (Fig. 2). So that it is indicated that Cav-1 function a vital regulator in MI.

Nonetheless, Cav-1 has been reported to participate in cellular survival and proliferation 31, 32, but the consensus has not been fully illustrated. Some studies established that Cav-1 induced fibrin fragment E internalization and triggered cellular apoptosis 33. But other investigators confirmed that Cav-1 could competitively interact with X-linked inhibitor of apoptosis (XIAP) which released endothelial NO synthase and increase the nitric oxide (NO) production which acts as the antiapoptotic effects 34. In our present study, we figured out that Cav-1 could sustain the cellular survival in ECs (Fig. 4). Additionally, Carlos Fernández-Hernando *et al.* have revealed that the loss of Cav-1 increased autophagy activation and alleviated the initiation of atherosclerosis in the aortic endothelium 35. In contrary to the previous finding, we found that knockdown of Cav-1 inhibits autophagy flux (Fig. 6), and β-CD pretreatment eliminates the effects of Cav-1 (Fig. 7). It is presumable that these studies highlight the endothelial LDL transcytosis in the presence of numerous oxidized lipids and proinflammatory cytokines which may be different patterns from the hypoxia injury.

In vascular endothelium, Cav-1 could maintain the cellular function by regulating of endothelial structure and permeability 36. Previous studies have demonstrated that the absence of Cav-1 can induce the endothelium hyper-permeability and inflammatory injury via altering the expression and assembly of junction proteins 36, 37. After the occurrence of ischemia, Cav-1 mediates the anti-inflammatory effects through suppressing the release of proinflammatory cytokine and the action of inflammatory signaling pathway, such as conventional NF-κB. Besides thrombo-contributed ischemia can induced the decrease of RXR-γ and intermediated the thrombo-inflammation in cerebral ischemia/reperfusion (I/R) injury 38. In agreement with these findings, we found that p65 was activated in disturbed Cav-1 groups under the hypoxia injury, which subsequently translocated into nuclear by analysis of the isolated nuclear and cytoplasmic lysates (Fig. 5). Additionally, studies have proved that the oxidative effects of Cav-1 depend on its phosphorylation 37. Richard D Minshall *et al.* have reported that the endothelial cadherin/β-catenin complexes were disassociated with the H_2_O_2_ stimulation, thereby inducing the endothelial barrier disruption, and deficiency of Cav-1 protects pulmonary vascular from H_2_O_2_ injury 37, 39. In contrast to the finding, our results indicated that the oxidative stress can be evoked after the downregulation of Cav-1 (Fig. 3). It is likely that the contributions by exogenous oxidants exposure to H_2_O_2_ and hypoxia stimulation were incommensurate when the endothelial Cav-1 proteins were disturbed 40, 41.

Cyclodextrin, an agent of cholesterol-depleting, has been reported to function by disrupting the caveolae structure and promotes caveolae scissile from the membrane 42. In our study, the Cav-1-siRNA mediated endothelial dysfunction was parallel with β-CD treatment groups suggesting that Cav-1 may preserves endothelial homeostasis through caveolae (Fig. 7). However, previous investigations have revealed that Cav-1 can significantly reduce NO production in ECs by inhibiting the eNOS activity 42, 43. And increased production of NO appeared to improve the dysfunction of endothelium. Thus, the exact role of NO in the Cav-1-mediated effects need to be further explored when it is suffered from ischemic insults.

Although our findings are promising, it is important to acknowledge that there are some limitations. Firstly, it is possible that additional mechanisms may contribute to the cardioprotective effects of Cav-1 in MI, and we have not fully elucidated the potential mechanisms. Besides, the current study did not include human specimens or treatments. Therefore, further researches are necessary to perform according to human contexts. Additionally, this study did not investigate the effects of Cav-1 in endothelial-cell specific knockout models which may not accurately reflect the specific role of Cav-1 in endothelial cells. Therefore, future researches by endothelial specific knockout models *in vivo* are necessary to detect the role of Cav-1 in MI.

In conclusion, our study addressed the importance of Cav-1 in maintaining the endothelial homeostasis following hypoxia insults and suggested the potential benefit of Cav-1 in hypoxia-related injury. Downregulation of Cav-1 increased infract zone and augmented the defection of heart function after MI injury, revealing that targeting Cav-1 may be a promising therapeutic strategy for MI. Prospective studies need to clarify which Cav-1-releated signaling pathway was involved into MI injury. Moreover, whether EC-Cav-1 is significant in the clinic still remains to be tested.

## Funding

This work was supported by the National Natural Science Foundation of China (Nos. 82372195&82172170); Tianjin Key Medical Discipline (Specialty) Construction Project (Nos. TJYXZDXK-054B) and the Natural Science Foundation of Tianjin (Nos. 21JCYBJC00250).
